# Effects of Extracorporeal Membrane Oxygenation Circuits on Drug Sequestration: A Review of Ex Vivo Experiments

**DOI:** 10.3390/jcm14228060

**Published:** 2025-11-13

**Authors:** Stéphane Bertin, David Haefliger, Antoine G. Schneider, Raphaël Giraud, Maria-Helena Perez, Xavier Bechtold, Ermindo R. Di Paolo, Laura E. Rothuizen, Thierry Buclin, Françoise Livio

**Affiliations:** 1 Service of Clinical Pharmacology, Lausanne University Hospital and University of Lausanne, 1011 Lausanne, Switzerland; 2 Adult Intensive Care Unit, Lausanne University Hospital and University of Lausanne, 1011 Lausanne, Switzerland; 3 Service of Intensive Care Medicine, University Hospital of Geneva, 1205 Geneva, Switzerland; 4 Pediatric Intensive Care Unit, Lausanne University Hospital and University of Lausanne, 1011 Lausanne, Switzerland; 5 Service of Cardiovascular Surgery, Lausanne University Hospital and University of Lausanne, 1011 Lausanne, Switzerland; 6 Service of Pharmacy, Lausanne University Hospital and University of Lausanne, 1011 Lausanne, Switzerland

**Keywords:** ex vivo model, extracorporeal membrane oxygenation (ECMO), sequestration, drug, medicine, pharmacokinetics (PK), logP, lipophilicity

## Abstract

**Background:** Extracorporeal membrane oxygenation (ECMO) can affect the disposition of drugs, notably by sequestering them in a circuit. This review aimed to provide a comprehensive summary of existing ex vivo studies investigating the impact of contemporary ECMO circuits on drug sequestration, and to examine the associations between the physicochemical properties of drugs, the features and settings of ECMO devices, and the extent of drug sequestration. **Method:** A comprehensive search was conducted to identify ex vivo studies that determined drug concentrations in ECMO circuits. Studies that did not allow for the proper assessment of drug loss by degradation were excluded. Drug characteristics and experimental conditions were recorded. Drug sequestration in the circuit was calculated as the difference between the drug loss measured in the ECMO circuit and the drug loss due to spontaneous degradation measured under control conditions. To identify predictors of drug sequestration, a stepwise multiple linear meta-regression was applied by testing the physicochemical properties of drugs and ECMO device features/settings. **Results:** A total of 40 studies were identified, of which 21 were included in the analysis, covering 41 drugs. The Maquet membrane oxygenator was the most used brand (73%). About half of the circuits were adult and half were pediatric. Our final regression model retained lipophilicity, and to a lesser extent ionization at a physiological pH, as significant predictors of drug sequestration (R^2^ 0.44, relative standard error 23%). Protein binding had no additional effect. Anti-infectives were the most studied class of drugs (n = 28). Antibiotics were overall not significantly sequestered, while lipophilic drugs such as posaconazole, voriconazole, paracetamol, fentanyl, sufentanil, propofol, thiopental, dexmedetomidine and amiodarone were highly sequestered (≥50%). However, this sequestration occurred mainly within the first few hours of the experiments, possibly reflecting a saturation effect. **Conclusions:** Lipophilic drugs are significantly sequestered in ex vivo ECMO circuits, although this effect may be limited by early saturation.

## 1. Background

Extracorporeal membrane oxygenation (ECMO) is a temporary life support modality used in critically ill patients with potentially reversible cardiac or respiratory failure that is unresponsive to conventional management [1,2]. ECMO circuits include cannulas, tubings, a centrifugal pump and a membrane oxygenator. Blood is drained from the patient’s venous circulation, pumped through the membrane oxygenator, and returned oxygenated and decarboxylated to either the venous circulation (veno-venous ECMO), thus providing pulmonary support, or the arterial circulation (veno-arterial ECMO), for both cardiac and pulmonary support [3]. Since the first clinical application of ECMO in the 1970s, there have been considerable technical improvements in pumps, membrane oxygenators and biocompatible coated surfaces, which led to safer and more effective ECMO devices. Hence, the use of ECMO has gradually expanded over the past 15 years to include a wider range of indications [4,5,6,7,8,9,10,11]. In particular, the COVID-19 pandemic gave unprecedented impetus to the use of ECMO [12].

Drug prescribing in critically ill patients is particularly challenging as acute conditions induce pathophysiological changes that cause significant alterations in drug pharmacokinetics (PK). ECMO may further affect PK parameters due to an expanded circulating volume (the addition of fluids to prime the extracorporeal circuit), altered hemodynamics induced by the ECMO system itself, systemic inflammation triggered by the exposure of blood to exogenous tubings and membrane oxygenator surfaces, and drug sequestration in the ECMO circuit, which altogether tend to affect drug volume of distribution (Vd) and clearance (CL).

The propensity for a drug to be sequestered in an ECMO circuit is influenced by its physicochemical properties. Lipophilic and highly protein-bound drugs appear more likely to be sequestered, but knowledge in this area remains limited [13,14]. The circuit component materials may also influence the extent of drug sequestration [15]. Oxygenators and tubings are now commonly coated with biocompatible compounds, such as heparin, albumin, phosphorylcholine, poly2-methoxyethyl acrylate or sulfated/sulfonated polyethylene oxide, to minimize the inflammatory response to artificial surfaces. Compared with early silicone oxygenators, novel polymethylpentene polymer hollow fiber membrane oxygenators are also associated with a lower inflammatory response, besides other advantages [16]. These more biocompatible materials decrease the risk of clotting and may also reduce drug sequestration in the circuit.

Ex vivo studies are performed using isolated (closed-loop) ECMO circuits including tubings, a pump and an oxygenator for adult or pediatric patients. They focus solely on the interaction between drug and circuit components, allowing the specific assessment of the extent of drug sequestration in the circuit, separately from all other patient-related influences.

This review aims to summarize and discuss the published observations on the ex vivo effects of currently used ECMO circuits on drug sequestration. It further aims to develop a predictive model of drug sequestration by exploring the correlations between the physicochemical properties of drugs as well as ECMO characteristics, and sequestration. To the best of our knowledge, this is the first review specifically focusing on ex vivo ECMO experiments.

## 2. Methods

### 2.1. Data Sources and Search Strategy

One author conducted a comprehensive literature search in PubMed and Embase to identify ex vivo studies published between January 1990 and March 2023, using the following terms: “ECMO ex vivo”, “ECMO ex vivo pharmacokinetics”, “extracorporeal membrane oxygenation ex vivo pharmacokinetics”, “sequestration extracorporeal membrane oxygenation ex vivo”. In addition, the references of these articles were systematically screened for further studies on the subject. The search was limited to articles published in English, French and German.

### 2.2. Inclusion and Exclusion Criteria

**Figure 1:**
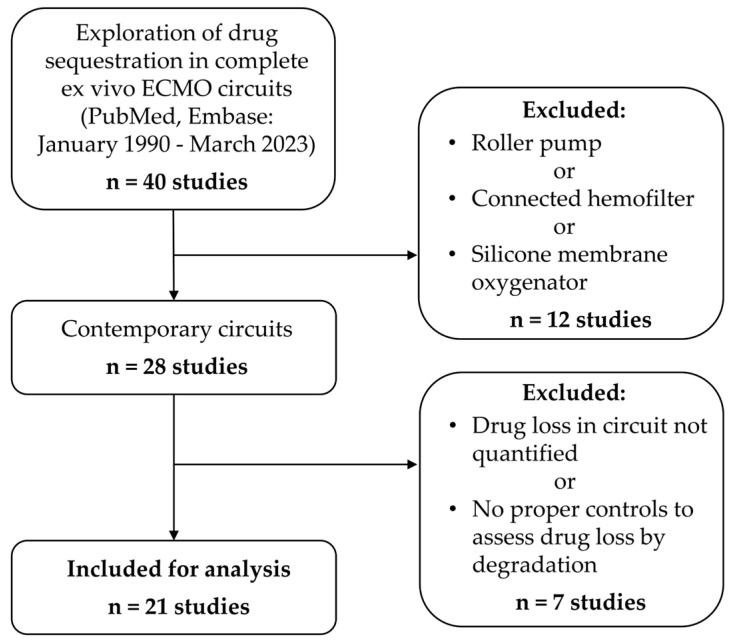
Review study screening and selection process.

We included all original ex vivo studies providing a determination of any drug concentration from a complete ECMO circuit, with an oxygenator, a pump and tubings. We excluded studies on cardiopulmonary bypass, with a hemofilter connected to the ECMO circuit, or on the extraction of non-drug products (i.e., contrast media, endogenous substances, xenobiotics). We also excluded studies using non-contemporary ECMO circuits, i.e., those with a silicone membrane oxygenator and/or a roller pump, and studies whose design did not allow for the adequate assessment of drug loss by spontaneous degradation (Figure 1). Drug loss by spontaneous degradation was considered assessable when drug concentrations were simultaneously measured in controls, i.e., from separate tubes that contained the drug at the same concentration as in the ECMO circuit at the start of the experiment. If the study did not include controls, or included controls without reporting their drug concentrations, drug loss was considered assessable only for samples taken from the ECMO circuit up to 6 h from the start of the experiment, assuming that the drugs tested are stable in blood at room temperature for at least 6 h [17]. Thus, in the absence of a control, we considered a loss of drug in the ECMO circuit during the first 6 h of an experiment as resulting from sequestration rather than spontaneous degradation. If drug loss in the circuit was quantified in only part of the study experiments, the study was included, but only the exploitable part was analyzed for drug sequestration. Two authors assessed all screened studies for eligibility and subsequently reviewed and analyzed the included studies.

### 2.3. Data Collection

We recorded the following characteristics for each drug studied: international non-proprietary name and drug class; drug lipophilicity expressed as logP (i.e., the logarithm of the n-octanol-water partition coefficient) [18]; logD (logarithm of the n-octanol-water partition at a given pH) at physiological pH of 7.4; protein binding (PB); molecular weight (MW); total polar surface area (TPSA); and ionization at physiological pH. The values of logP, logD, PB, MW, TPSA and ionization at physiological pH were drawn from the SwissADME [19], DrugBank [20] and ChEMBL [21] databases. The logP value can differ depending on the sources consulted, hence an average value extracted from SwissADME [19] was retained. There was less inter-source discrepancy regarding the other physicochemical properties of drugs. We documented the circuit components: model type and surface of oxygenator; tubings and pump and their respective coatings. We recorded the experimental conditions: type of matrix (blood, crystalloid); circuit volume and flow rate; temperature; pH; oxygenation and gas balance; albumin and electrolyte concentrations; anticoagulation; number of different drugs injected in the same ECMO run; presence or absence of controls; and study duration and number of samples collected. Drug sequestration in the circuit was calculated by subtracting drug loss in the control samples (assumed to result from degradation) from drug loss in the circuit at the end of the run, or earlier if subsequent data were insufficient to quantify drug sequestration. Drugs were considered highly sequestered if sequestration was ≥50%, and moderately sequestered if sequestration was >30% and <50%. Sequestration ≤ 30% was considered low, as it falls within the range of the lowest interindividual PK variability values (10–30%) observed in clinical studies. When different types of experiments with a given drug were carried out within the same study, we reported their results individually. In the case of replicated experiments under the same conditions, we reported the results as a single experiment (mean value across replications).

### 2.4. Descriptive Data Analysis

We summarized all of the following recorded data with Microsoft Excel: list of drugs studied along with their respective ATC (Anatomical Therapeutic Chemical) codes [22]; pharmacological/therapeutic subgroups; physicochemical properties of drugs; number of different experiments and studies for each drug; oxygenator brands used; proportions of adult and pediatric ECMO circuits; oxygenator’s coating; gas balance; albumin concentration; number of different drugs injected in the same ECMO run; duration of the runs; number of drug samplings per study; and estimates of drug sequestration in the circuit, including median and range if ≥4 different experiments for a single drug were performed. The results were expressed as a percentage or the median with a range.

### 2.5. Statistical Analysis and Model Building

To identify factors predictive of drug sequestration in ECMO circuits, we applied a stepwise multiple linear meta-regression including the physicochemical properties of the drugs and ECMO device features and settings. First, we performed sequential univariable regressions across the articles, relating the percentage of sequestration with logP, logD, TPSA, PB, MW, ionization at physiological pH, oxygenator surface, number of different drugs injected in the same ECMO run and number of matrix passages through the oxygenator (defined as matrix flow rate divided by volume in the experiment). TPSA and MW were log-transformed to normalize their distribution. We built up a multivariable model by the stepwise forward selection of predictors, using a *p*-value < 0.1 as the inclusion criterion. Finally, we verified this meta-regression by also performing a backward deletion of predictors, retaining only those significant at a *p*-value < 0.05, to check convergence with the forward approach. We evaluated the final model graphically by plotting the observations against the predictions, the residuals against the fitted values, the QQ (quantile–quantile) plot of residuals and the Cook’s distance values. We checked the multicollinearity between predictors in the final model with the variance inflation factor score. We assessed the model performance with the linear regression’s coefficient of determination (R^2^), R^2^ adjusted for the number of predictors and relative standard error (RSE). The modeling was performed with R version 4.3.2 software [23].

## 3. Results

### 3.1. Data Description

Our literature search retrieved 40 studies. After applying the exclusion criteria, we included 21 studies for analysis (Figure 1), which covered 41 drugs from eight different second-level ATC pharmacological/therapeutic subgroups. Anti-infectives were the most studied class of drugs (n = 28), notably antibacterials (n = 21) [14,24,25,26,27,28,29,30,31,32,33,34,35,36]. Nervous system drugs came second (n = 12) with analgesics (n = 3), general anesthetics (n = 3), antiepileptics (n = 3) and psycholeptics (n = 3) [14,28,29,31,37,38,39,40,41]. Amiodarone was the only cardiovascular drug studied [42,43]. The number of studies varied for each drug (median 1, min 1, max 4). The most studied drugs were meropenem, vancomycin, caspofungin, voriconazole, fentanyl, sufentanil, morphine, paracetamol and midazolam (Table 1).

The Maquet membrane oxygenator was the most frequently used brand in these studies (73%); the other brands were Xenios (9%), Sorin (9%), Medos (4.5%) and Chalice (4.5%). Pediatric and adult circuits were used in 38% and 40% of the experiments, respectively; there was no information for the remaining 22%. Most of the circuits were filled with blood (blood 93%, crystalloids 7%). The type of oxygenator coating was reported in 17 studies (81%), gas balance in 5 (24%) and albumin concentration in 4 (19%). In 14/21 studies, more than one drug was injected during the same ECMO run. The median number of different drugs injected in the same ECMO run was 2 (min 1–max 13). The median duration of the studies was 24 h (min 2–max 72 h) (Table 2). The median number of samplings per study was 9 (min 3–max 24). The drug sequestration of the 41 drugs studied is presented in Table 1. The drugs that were highly sequestered (≥50%) were posaconazole (52%), voriconazole (80%, 7–82%), paracetamol (53%, 43–59%), fentanyl (80%, 24–90%), sufentanil (88%, 62–94%), propofol (86%, 89%), thiopental (90%), dexmedetomidine (61%) and amiodarone (59%, 99%). Notably, 50% sequestration was reached within 3 h from the start of the experiments for most of these drugs [14,26,28,29,31,38,41,42,43]. The drugs that were moderately sequestered (>30%) were vancomycin (35%, 9–50%), teicoplanin (3%, 60%), morphine (49%, −6–67%), lorazepam (40%) and midazolam (43%, 35–87%). There was notable variability in the sequestration values for the same drugs across different studies, with a mean of relative variability and extreme values of 122% (32–249%). Negative sequestration values, indicating greater drug loss in controls than in ECMO circuits, were observed in only a few studies and involved drugs with low sequestration.

### 3.2. Factors Affecting Sequestration

LogP, logD, TPSA and PB were identified as significant predictors of drug sequestration using univariate analysis. Our final regression model, obtained using the forward selection of predictors, retained both logP and ionization at physiological pH as significant predictors for the percentage of drug sequestration, with *p* = 1.07·10^−12^ and *p* = 0.03, respectively (Supplemental Table S1). All other predictors tested had a negligible magnitude of effect and *p*-values > 0.1; therefore they were not retained. Among the model’s predictors, logP had the highest coefficient (+8.2% per logP unit) and thus the greatest impact on drug sequestration. Of note, the backward deletion procedure retained the same predictors but identified further factors as statistically significant predictors, notably the number of different drugs injected in the same ECMO run and the number of matrix passages through the oxygenator (*p* < 0.05). However, their coefficients (−2% and +2.3% per unit, respectively) were low, thus their impact on drug loss was considered negligible. Therefore, we did not retain them in the model. The final model equation is

Drug sequestration (%) = 27.8 + 8.2 · logP + 4.8·charge

The model’s coefficient of determination (R^2^) is 0.44 (adjusted R^2^ 0.42), with a residual standard error of 23.6% (Supplemental Figure S1). The slightly bowed shape of the smoothed scatter plot of residuals versus fitted values seems acceptable, indicating a rather constant variance. The normality of the residuals is confirmed in the Q-Q plot. Some outliers were observed, but the Cook’s distance was less than 0.5 for each observation, meaning that these values do not negatively affect the model (Supplemental Figure S2). Variance inflation factor values equal 1 for both predictors, excluding significant collinearity (Supplemental Figure S3). Table 3 presents the coefficient estimates with standard errors and *p*-values for the multiple linear regression, along with the performance of the model.

## 4. Discussion

Our review confirms that lipophilic drugs are highly sequestered by ECMO circuits. Lipophilicity is the main independent predictor of drug sequestration in ECMO circuits according to our model. Drugs with higher logP values are more susceptible to sequestration, likely because their hydrophobic regions interact with the hydrophobic polymeric components of tubing and oxygenator membranes, such as PVC or polymethylpentene [44]. Although coatings increase surface hydrophilicity and reduce hydrophobic interactions, ex vivo data suggest that the adsorption of highly lipophilic compounds still occurs. Consequently, lipophilicity remains the main determinant of circuit-related drug sequestration. In contrast, PB, known to correlate positively with logP, had no significant impact. This differs from an ex vivo study that looked at 13 drugs, where both logP and PB predicted the amount of sequestration [14]. The intercorrelation between both factors probably explains that either one or the other or both can be retained as significant predictors, depending on the particularities of the datasets studied. A similar situation is likely to exist with logD. The ionization at physiological pH was the other independent predictor affecting sequestration, although to a much lesser extent. This can be interpreted as reflecting the higher affinity of cationic molecules for the commercial ECMO circuits used in ex vivo studies, which are commonly treated with anionic coating agents [45]. Given an R^2^ of 44% and an RSE of 23%, our model is not able to give precise predictions of drug sequestration in ECMO circuits. Nevertheless, it may provide a rough estimate of drug sequestration levels in ex vivo ECMO circuits when no data are available, such as for levosimendan, an inotropic agent used to assist in weaning from veno-arterial ECMO. Based on its logP (2.4) and ionization degree at physiological pH (−1), our model predicts a 42% sequestration of levosimendan, which is in reasonable agreement with the 33% observed in an ex vivo ECMO experiment (unpublished data, “Sequestration of levosimendan and its metabolites, OR-1855 and OR-1896, on extracorporeal membrane oxygenation circuits: an ex vivo study”, abstract No. 286 presented at the French Society of Anesthesia and Intensive Care Medicine (SFAR) 2024 Congress, Paris, France).

Anti-infectives were the most studied drug class in ex vivo ECMO circuits. Antibacterials, which are predominantly hydrophilic, appear to be overall not significantly sequestered in ex vivo ECMO circuits (<30%), with the notable exceptions of glycopeptides, vancomycin and teicoplanin. Median vancomycin sequestration was 35% (range 9–50%) [26,29,31]. In a single study, teicoplanin sequestration varied according to the oxygenator, i.e., 60% and 3% sequestration with the Sorin and Maquet oxygenators, respectively [30].

In prospective clinical studies in adults, the PK of antibacterials commonly used in intensive care, such as cefepime, piperacillin/tazobactam, meropenem, ceftriaxone, amikacin, ciprofloxacin and even vancomycin, were similar between patients on ECMO and other critically ill patients without ECMO [46,47,48,49,50,51,52,53,54]. Clinical data are insufficient to assess the impact of ECMO on teicoplanin PK [55]. Thus, overall, clinical studies on antibacterials tend to be broadly consistent with ex vivo studies.

Antifungals were less often studied than antibacterials. Highly lipophilic antifungals were significantly sequestered in ex vivo circuits: median voriconazole and posaconazole sequestration were 80% (range 7–82%) and 52%, respectively [26,34]. Poorly lipophilic or hydrophilic antifungals were less sequestered: fluconazole sequestration was 2–11% and micafungin 14–31%, while median caspofungin sequestration was 10% (range 1–43%) [14,27,30,34]. There was no ex vivo ECMO data on amphotericin B, isavuconazole or itraconazole.

Data regarding voriconazole PK in adult patients on ECMO are scarce and no robust prospective clinical PK studies were found. However, most available clinical data suggest that voriconazole undergoes some degree of sequestration, which may indicate that adjustments are needed in the dosing of this antifungal [56,57,58,59,60]. In a unique study on posaconazole, PK parameters in ECMO patients did not substantially differ from those in non-intensive care unit (ICU) hematological patients [61]. Fluconazole and micafungin PK data in adult patients on ECMO are scarce. A population PK study including six patients on ECMO and continuous renal replacement therapy (CRRT) showed that fluconazole therapeutic concentration targets were not achieved; however, these observations were primarily attributed to high CRRT dialytic CL rather than sequestration by ECMO circuits [62]. In one case report, the ECMO circuit had no significant effect on the exposure to fluconazole [63]. The PK parameters of micafungin and caspofungin were not significantly different in patients on ECMO compared with those without ECMO [60,64,65,66].

Ex vivo ECMO studies on antivirals were limited to remdesivir, which is approved for the treatment of COVID-19. Remdesivir is a prodrug and GS-441524 is one of its main active metabolites. It has a short half-life (1 h) and is poorly lipophilic, whereas GS-441524 has a longer half-life (27 h) and is hydrophilic. Neither remdesivir nor GS-441524 were significantly sequestered in ex vivo ECMO circuits [33]. The PK of GS-441524 was also not shown to be affected by ECMO in patients [67].

The second most studied class of drugs in ex vivo ECMO circuits was central nervous system (CNS) drugs, including analgesics, anesthetics, psycholeptics and antiepileptics [14,28,29,31,37,38,39,40,41]. These lipophilic drugs were either highly or moderately sequestered in ex vivo ECMO circuits, except for the less lipophilic antiepileptics.

In a population PK study in adults on ECMO, sufentanil CL was lower than that reported by another study in ICU patients not on ECMO (37 L/h versus 56 L/h) [68,69]. However, the latter involved patients that were less severely ill and notably without liver function impairment, which may, independently of ECMO, explain the difference in CL. Fentanyl and propofol PK were not affected by ECMO, except for a transient increase in CL just after ECMO initiation. According to the authors, this transient increase in apparent CL was mainly caused by dilution, alongside saturable drug sequestration in the ECMO circuit [70].

In an exploratory PK study in pediatric patients on ECMO, observed dexmedetomidine concentrations were compared with predicted concentrations using published PK models in critically ill infants not on ECMO. Most of the evaluated models overpredicted dexmedetomidine plasma concentrations, potentially indicating increased CL on ECMO [71]. A study investigating the dose–concentration relationship of midazolam in neonates on ECMO revealed measured concentrations less than half those predicted. Of note, this was only observed during the first 18 h after ECMO initiation, likely due to an increased Vd by the circuit volume and saturable sequestration [72]. Two studies on midazolam PK in neonates on ECMO found an increased Vd, which is expected given the high circuit-to-blood volume ratio in these patients [73,74].

Levetiracetam (low lipophilicity) and phenytoin were not significantly sequestered in ex vivo ECMO circuits. This appears to be consistent with clinical data, although limited to case reports [75,76,77].

There are no published PK observations in patients on ECMO for morphine, paracetamol, thiopental, lorazepam and lacosamide.

Amiodarone, the only treatment intended for cardiac purposes studied in ex vivo ECMO circuits [43], was shown to be highly sequestered. Clinical data are limited to one case report suggesting that larger doses of amiodarone may be required to achieve a therapeutic effect in neonates on ECMO [78]. According to a physiologically based pharmacokinetic (PBPK) model parametrized with ex vivo ECMO data [43], a higher loading dose of amiodarone is recommended to treat cardiac arrest with ventricular arrhythmia in pediatric patients on ECMO than in those not on ECMO (22 mg/kg in neonates, 13 mg/kg in infants, 8 mg/kg in children on ECMO versus a 5 mg/kg standard dose for those not on ECMO) [79,80]. Given that loading dose is primarily determined by Vd, the need for a higher initial dose of amiodarone appears to be predominantly driven by the additional volume introduced by the ECMO circuit. This is further supported by the fact that, according to the PBPK model, the proposed weight-based dose is highest in neonates, who have the greatest circuit-to-blood volume ratio.

Thus, considering the available evidence, while extensive sequestration of highly lipophilic drugs is observed in ex vivo ECMO circuits, this sequestration does not appear to consistently affect the PK of these drugs in clinical studies involving ECMO patients. In clinical practice, if PK alterations related to ECMO are suspected, therapeutic drug monitoring, when available, may help ensure adequate drug exposure.

## 5. Limitations

The limited number of studies available for inclusion and the substantial methodological heterogeneity represent the main limitations of this review.

The methodological heterogeneity across the studies likely explains some of the conflicting results regarding drug sequestration, such as those reported for vancomycin (median 35%, range 9–50%) or sufentanil (median 88%, range 62–94%). Sources of heterogeneity primarily involved oxygenator models, tubing coatings, gas sweeping, circuit volume, flow rate, drug dose and the number of drugs tested per run, all of which can influence drug sequestration. In addition, a significant proportion of the studies did not thoroughly report procedural details: for instance, albumin level or gas sweeping were rarely reported, even though both parameters can impact the sequestration of drugs.

More than one drug was injected in the same ECMO run in approximately two-thirds of the included studies. This has the advantage of mimicking real-life conditions, since patients on ECMO typically receive numerous drugs, but it makes it difficult to estimate the net sequestration of one specific drug. There is evidence suggesting that ECMO circuits can become rapidly saturated over time, and saturation could occur even earlier if multiple drugs compete for sequestration. In the studies reviewed, drug sequestration occurred during the initial hours of the experiments, with minimal or no sequestration thereafter. This suggests that the binding sites on the circuit were quickly occupied and became saturated [14,26,28,29,31,38,41,42,43]. In one of the latter studies, propofol was highly sequestered shortly after a bolus, but no further significant sequestration was observed during a subsequent continuous infusion, highlighting a saturation effect [41]. In addition, the studies included in this review report relative sequestration values, which may be misleading when assessing clinical relevance. Ex vivo ECMO circuits have indeed a limited circulating volume (typically <1 L), meaning that the drug doses required to achieve “therapeutic” concentrations are much lower than those administered in clinical practice. As a result, the absolute amount of sequestered drug relative to the overall intended treatment course might be low in real-life settings, even if the relative sequestration rate is high in ex vivo experiments. Therefore, ECMO may have a limited effect on drug CL in patients, and consequently on the drug dose regimen, since CL is the primary determinant of the maintenance dose.

Another aspect not captured by ex vivo ECMO models is that they do not account for the increased Vd caused by the extracorporeal circuit. The effect of the ECMO circuit on Vd is particularly pronounced in neonates and young children, due to their elevated circuit-to-blood volume ratio compared with adults. This is a crucial factor to consider in clinical practice, as Vd is the primary determinant for the loading dose.

## 6. Conclusions

Ex vivo models offer a valuable approach for investigating drug sequestration in ECMO circuits. However, significant heterogeneity exists in both study design and reporting, which complicates the interpretation of the published observations. Consequently, there is a need to standardize the performance and reporting of these complex experiments, with respect to both the methods and results.

Although lipophilicity, and to a lesser extent ionization, were identified as key determinants of drug sequestration in ECMO circuits, these findings cannot be directly extrapolated to clinical settings. Ex vivo models do not fully replicate the complexity of in vivo conditions, and drugs showing extensive sequestration in these systems may not undergo the same extent of sequestration in patients. This discrepancy may in part be explained by the rapid saturation of circuit binding sites by the drug. Moreover, ex vivo studies do not account for ECMO-related increases in Vd, which are particularly relevant in neonates and young children and can significantly influence loading dose requirements.

While ex vivo ECMO studies cannot fully capture the complexity of PK in real-life conditions, data from these experiments can be used to parameterize PBPK models to specifically account for drug interactions with ECMO components. PBPK modeling has become an increasingly valuable technique for predicting PK in various clinical conditions, including ECMO [79,81,82,83]. These complex models can help generate preliminary dosing recommendations, which should ideally be validated in clinical PK studies, with a limited number of patients being typically sufficient.

## Figures and Tables

**Table 1:** Physicochemical properties of drugs and extent of sequestration in ex vivo extracorporeal membrane oxygenation.

Drug	LogP	LogD	Protein Binding (%)	Molecular Weight (g/mol)	TPSA (Å^2^)	Ionization ^a^	Drug Sequestration ^b^ (%)	Study References
**Anti-infectives**								
**Antibacterials**								
amikacin	−5.91	−15.10	10	582.6	331.9	4	6	[32]
amoxicillin	−0.39	−2.67	17	365.4	158.3	0	−9	[24]
cefazolin	−0.15	−5.01	80	454.5	234.9	−1	2.1	[31]
cefepime	−2.58	−3.45	20	480.6	203.6	0	−6, 18.4	[24,35]
cefiderocol	−1.86	NA	50	752.2	310.4	−1	0	[36]
cefoperazone	−0.98	−4.35	87.5	645.7	270.9	−1	17, 23	[30]
cefotaxime	−0.24	−4.20	32.5	455.5	227.1	−1	18	[24]
ceftazidime	−1.39	−6.94	13.9	546.6	244.8	−1	−7	[24]
ceftolozane	−3.43	−7.80	18.5	666.7	355.8	0	2.9	[25]
ceftriaxone	−0.72	−5.53	95	554.6	293.8	−2	−9, 22	[14,24]
ciprofloxacin	1.10	−0.85	30	331.3	74.6	0	23	[14]
gentamicin	−2.15	NA	15	1390.7	199.7	5	1	[32]
linezolid	1.23	0.64	31	337.3	71.1	0	11	[14]
meropenem	−0.37	−4.36	2	383.5	135.5	0	21 (10.9–48)	[29,30,31]
oxacillin	1.85	−1.59	94.2	401.4	138.0	−1	−3	[24]
piperacillin	−0.24	−3.64	18.5	517.6	138.8	−1	−10	[24]
polymyxin B	−5.28	NA	85.5	1203.5	510.9	5	−1.56	[30]
sulbactam	−0.33	−4.35	38	233.2	100.1	−1	7, 20	[30]
tazobactam	−1.04	−4.89	30	300.3	130.8	−1	0.06	[25]
teicoplanin	−2.30	NA	92.5	1879.7	NA	1	3, 60	[30]
vancomycin	−3.10	NA	50	1449.3	NA	1	35.5 (9–50)	[26,29,31]
**Antifungals**								
caspofungin	0	NA	97	1093.3	NA	2	10.5 (1.9–43)	[14,30,34]
fluconazole	0.88	0.56	11.5	306.7	81.7	0	2, 11	[14,27]
micafungin	−1.50	NA	99	1270.3	NA	−1	14.2, 31.97	[27,30]
posaconazole	4.23	5.41	98	700.8	115.7	0	52.2	[34]
voriconazole	2.40	1.82	58	349.3	76.7	0	80.3 (7.8–82.2)	[26,34]
**Antivirals**								
GS-441524	−1.90	−1.88	2	291.3	NA	0	1.6	[33]
remdesivir	1.50	2.01	90.8	602.6	213.4	0	20.6	[33]
**Nervous system**								
**Analgesics**								
fentanyl	3.78	2.43	82.5	336.5	23.6	1	80 (24.1–90)	[29,31,38,39]
morphine	1.44	−0.60	35	285.3	52.9	1	49 (−6–67.9)	[29,31,38,39]
paracetamol	0.93	0.90	17.5	151.2	49.3	0	53 (43–59)	[31,38]
**Antiepileptics**								
lacosamide	0.85	−0.02	15	250.3	67.4	0	18.1, 20.5	[37]
levetiracetam	0.10	−0.59	10	170.2	63.4	0	7, 10.5	[37]
phenytoin	1.81	1.07	90	252.3	58.2	0	26.5, 29	[37]
**Anesthetics, general**								
propofol	3.36	4.16	97	178.3	20.2	0	86, 89	[28,41]
thiopental	1.88	2.38	80	242.3	90.3	−1	90	[14]
sufentanil	3.93	2.13	92	386.6	61.0	1	88 (62–94)	[38]
**Psycholeptics**								
dexmedetomidine	2.88	3.34	94	200.3	28.7	0	61	[40]
lorazepam	2.68	3.53	85	321.2	61.7	0	40.4	[39]
midazolam	3.61	3.95	97	325.8	30.2	0	43 (35–87)	[29,31,38,39]
**Cardiovascular system**								
**Cardiac therapy**								
amiodarone	6.49	6.10	96	645.3	42.7	1	59.1, 99.3	[42,43]

LogP: logarithm of the n-octanol-water partition coefficient; LogD: logarithm of the n-octanol-water partition coefficient at physiological pH of 7.4; TPSA: total polar surface area, expressed in Å^2^ (square angstroms). ^a^ Ionization at physiological pH; ^b^ if ≥4 experiments: median (min–max) percentage of drug sequestration is provided.

**Table 2:** Circuit characteristics and experimental conditions of included studies.

Oxygenator Brand	Oxygenator Model	Oxygenator Surface (m^2^)	Oxygenator Coating	Tubing Coating	Matrix	Volume Circuit (mL)	Flow Rate (L/min)	T (°C)	pH	Number of Drugs	Study Duration (h)	Controls	Study References
Maquet	PLS Quadrox D adult	1.8	Bioline	Bioline	blood	668	4.5	physiol.	7.2–7.6	13	24	yes	[14]
Maquet	Quadrox	NA	Bioline	Bioline	blood	900	3	physiol.	NA	7	48	yes	[24]
Sorin	EOS	1.2	ChoP	NA	blood	NA	2.8	physiol.	7.2–7.5	2	24	yes	[25]
Xenios	miniLung petite	0.32	Rheoparin	NA	blood	225	0.5	physiol.	7.3–7.5	2	24	yes	[26]
Xenios	miniLung	0.65	Rheoparin	NA	blood	280	0.7	physiol.	7.3–7.5	2	24	yes	[26]
Xenios	ILA active	1.3	Rheoparin	NA	blood	360	2.5	physiol.	7.3–7.5	2	24	yes	[26]
Xenios	XLung	1.9	Rheoparin	NA	blood	400	3.5	physiol.	7.3–7.5	2	24	yes	[26]
Maquet	Quadrox iD adult or pediatric	1.8 or 0.8	Bioline	ChoP	blood	NA	1	physiol.	7.2–7.5	1	24	yes	[27]
Maquet	Quadrox	NA	Bioline	Bioline	blood	800	4.5	physiol.	NA	4	48	yes	[28]
Maquet	PLS Quadrox D adult	1.8	Bioline	Bioline	blood	668	4.5	physiol.	7.3–7.6	13	24	yes	[29]
Maquet	PLS Quadrox adult	1.8	Bioline	Bioline	blood	818	4.5	physiol.	7.2–7.5	7	24	yes	[30]
Sorin	D905 EOS ECMO	1.2	ChoP	ChoP	blood	525	4.5	physiol.	7.2–7.5	9	24	yes	[30]
Medos Hilite	800LT	0.32	Rheoparin	coated	blood	200	0.4	RT	physiol.	7	3	no	[31]
Maquet	HLS advanced 7.0	1.8	Bioline	Bioline	crystalloid	5351	3	RT	7.23	2	6	no	[32]
Maquet	Quadrox iD adult	1.8	Bioline	Smart-X	blood	1200	1	physiol.	7.2–7.5	2	12	yes	[33]
Chalice	paragon	NA	Rheopak	Carmeda	blood	700–750	4.5	physiol.	7.3–7.5	1	24	yes	[34]
Maquet	Quadrox iD adult	1.8	Softline	Smart-X	blood	1000	1	physiol.	7.2–7.5	1	24	yes	[35]
Maquet	Quadrox i adult	1.8	NA	NA	blood	NA	4.5	physiol.	NA	1	24	yes	[36]
Maquet	Quadrox i neonatal	0.38	NA	NA	crystalloid	400	1.5	physiol.	7.4–7.5	3	24	yes	[37]
Maquet	Quadrox i neonatal	0.38	NA	NA	blood	400	1.5	physiol.	7.4–7.5	3	24	yes	[37]
Xenios	miniLung petite	0.32	Rheoparin	NA	blood	225	0.5	physiol.	7.3–7.5	5	24	yes	[38]
Xenios	miniLung	0.65	Rheoparin	NA	blood	280	0.7	physiol.	7.3–7.5	5	24	yes	[38]
Xenios	ILA active	1.3	Rheoparin	NA	blood	360	2.5	physiol.	7.3–7.5	5	24	yes	[38]
Xenios	XLung	1.9	Rheoparin	NA	blood	400	3.5	physiol.	7.3–7.5	5	24	yes	[38]
Maquet	Quadrox D	1.8	NA	Carmeda	blood	NA	1	physiol.	NA	4	48	yes	[39]
Maquet	Quadrox iD adult	1.8	Bioline	ChoP	blood	NA	1	physiol.	7.2–7.5	1	24	yes	[40]
Maquet	Quadrox iD	NA	Bioline	Cortiva BioActive	blood	550	1	physiol.	physiol.	2	10	yes	[41]
Maquet	PLS-I	1.8	Bioline	NA	crystalloid	770	3.5	physiol.	NA	1	2	yes	[42]
Maquet	Quadrox iD adult	1.8	NA	ChoP	blood	NA	1	physiol.	7.2–7.5	1	72	yes	[43]

ChoP: phosphorylcholine; NA: not available; T: temperature; physiol.: physiologic; RT: room temperature.

**Table 3:** Meta-regression model for the prediction of drug sequestration in ECMO: coefficient estimates and performance evaluation.

Coefficients				Global Model			
Variable	Estimate	Standard Error	*p*-Value	R^2^	Adjusted-R^2^	RSE	*p*-Value
LogP	+8.2	1.0	1.07·10^−12^	0.44	0.42	23.59	6.1·10^−12^
Ionization	+4.8	2.2	0.03				

LogP: logarithm of the n-octanol-water partition coefficient (coefficient as percent of sequestration per LogP unit); Ionization: at physiological pH (coefficient as percent of sequestration per ionization unit); R^2^: coefficient of determination; RSE: relative standard error (as percent of sequestration).

## Data Availability

The datasets used and analyzed during the current study are available from the corresponding author on reasonable request.

## Supplementary Materials

The following supporting information can be downloaded at: https://www.mdpi.com/article/10.3390/jcm14228060/s1. Table S1: Predictors’ effect on drug sequestration in univariate and multivariate analysis. Figure S1: Predicted versus observed values of drug sequestration in ECMO circuits based on a meta-regression model. Figure S2: Meta-regression model diagnostic plots. Figure S3: Correlation matrix of predictor variables in meta-regression analysis.

## List of Abbreviations

ATC	anatomical therapeutic chemical
CNS	central nervous system
CL	clearance
CRRT	continuous renal replacement therapy
Vd	volume of distribution
ECMO	extracorporeal membrane oxygenation
ICU	intensive care unit
LogP	logarithm of the n-octanol-water partition coefficient
LogD	logarithm of the n-octanol-water partition coefficient at a given pH
MW	molecular weight
PB	protein binding
PBPK	physiologically based pharmacokinetic
PK	pharmacokinetics
QQ	quantile–quantile
RSE	relative standard error
TPSA	total polar surface area
