# Supplementary material for: Effects of Extracorporeal Membrane Oxygenation Circuits on Drug Sequestration: A Review of Ex Vivo Experiments
Source: J Clin Med. 2025 Nov 13;14(22):8060. doi: 10.3390/jcm14228060 (PMC12653448; doi:10.3390/jcm14228060)
Supplement: Supplementary file 1 [file jcm-14-08060-s001.zip › jcm-3945548-supplementary.pdf]

Supplemental Table S1: Predictors' effect on drug sequestration in univariate and multivariate analysis.

	Univariate analysis[a]		Multivariate analysis[b]	
**Predictor**	**R^2**	**p-value**	**R^2**	**p-value**
LogP	0.41	$6.43*10^{-12}$	NA	NA
Total polar surface area	0.42	$1.34*10^{-10}$	0.51	0.12
LogD	0.41	$3.62*10^{-10}$	0.48	0.9
Protein binding	0.13	$3.00*10^{-4}$	0.42	0.25
Molecular weight	0.07	0.01	0.41	0.33
Oxygenator surface	0.02	0.20	0.43	0.21
Number of drugs injected in the same ECMO run	0.01	0.34	0.41	0.57
Number of matrix passages in the oxygenator	0.001	0.35	0.45	0.27
Ionization	0.007	0.41	0.44	0.03

[a]Predictors tested one at a time. [b]Addition of one predictor to LogP (2 predictors tested together).

LogP: logarithm of the n-octanol-water partition coefficient; LogD: logarithm of the n-octanol-water partition at physiological pH of 7.4; Number of matrix passages in the oxygenator: defined as matrix flow rate divided by volume in the ECMO circuit; ionization: molecular charge at physiological pH; NA: not applicable; R^2: coefficient of determination.

Supplemental Figure S1 Predicted versus observed values of drug sequestration (%) in ECMO circuits based on meta-regression model.

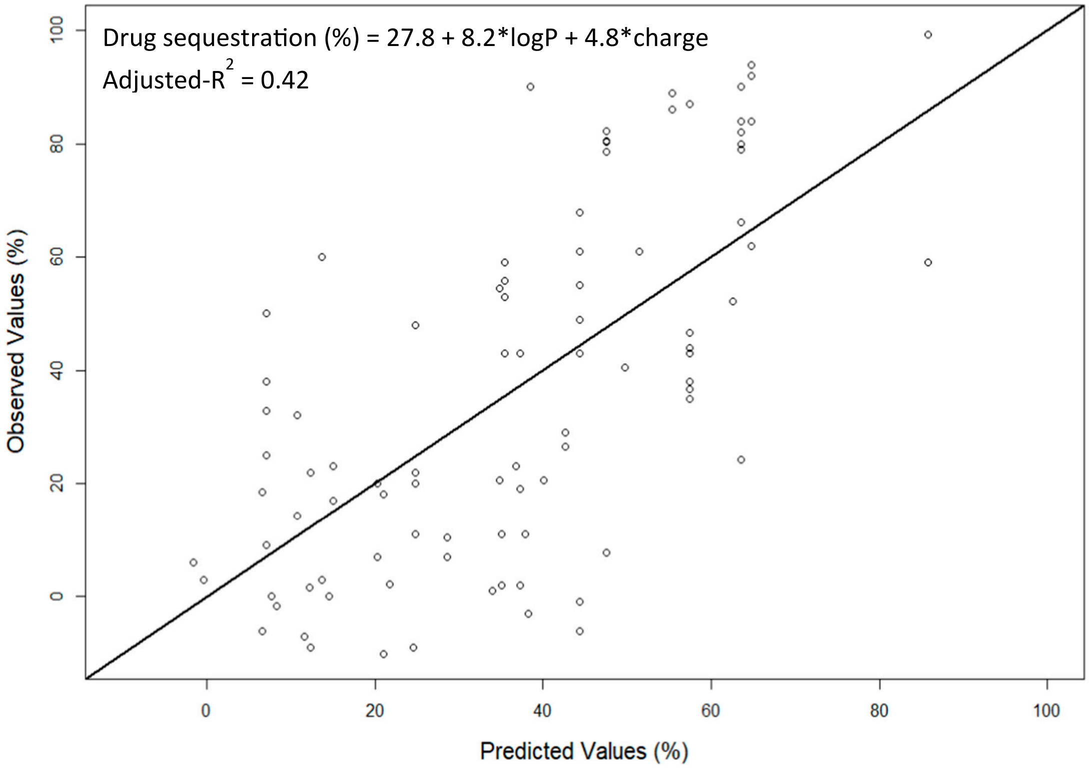

Supplemental Figure S2: Meta-regression model diagnostic plots.

A

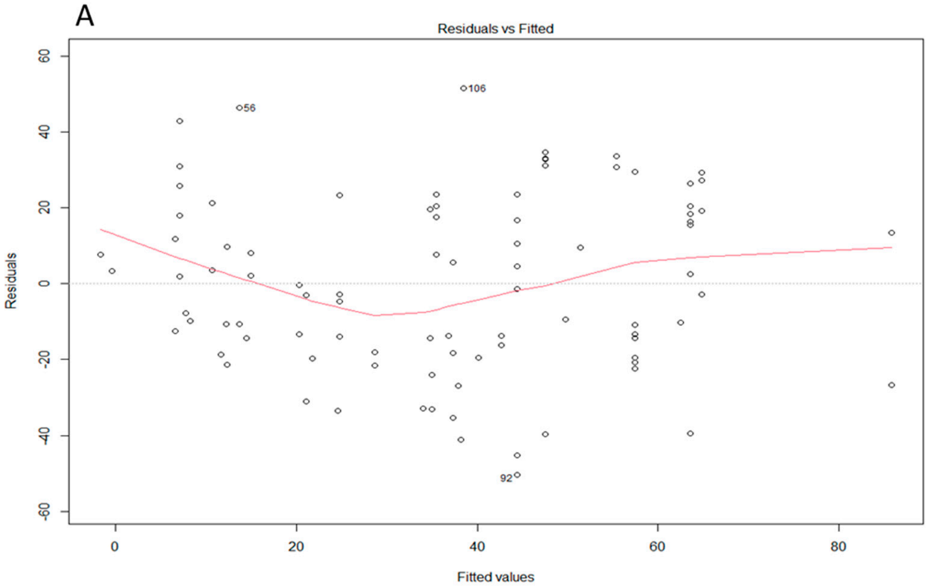

B

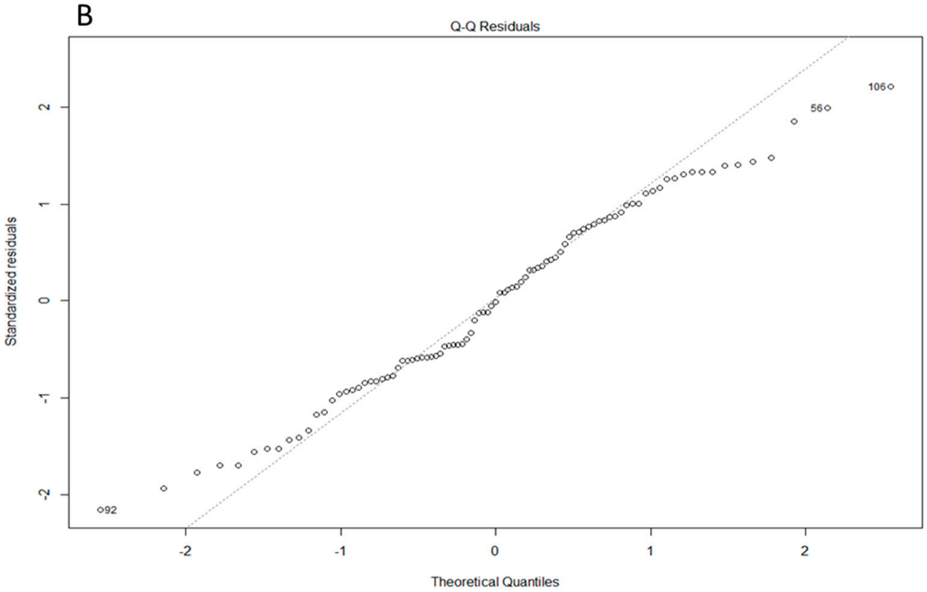

A: Scatter plot of residuals versus predictions.

B: Quantile-quantile plot of standardized residuals along theoretical normal deviation.

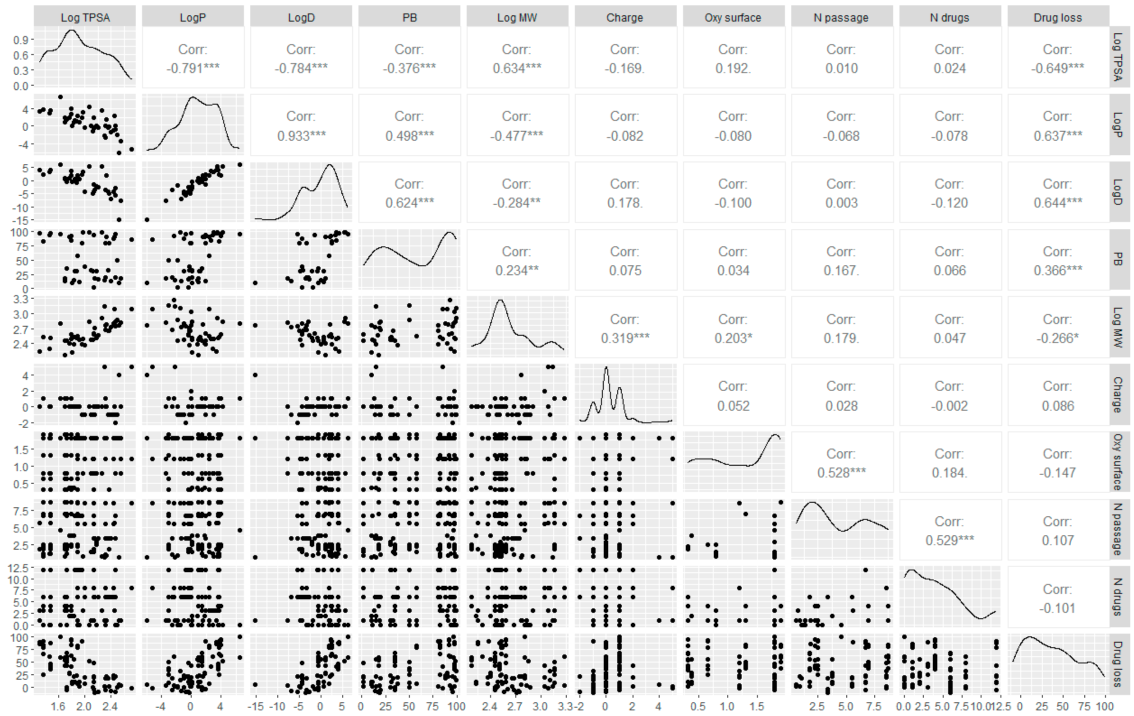

Supplemental Figure S3: Correlation matrix of predictor variables in meta-regression analysis. Intersection of pairs of predictors show their graphical association and the corresponding r value with significance level: *p<0.05; **p<0.01; ***p<0.001.

Log TPSA: logarithm of total polar surface area; LogP: logarithm of the n-octanol-water partition coefficient; LogD: logarithm of the n-octanol-water partition at physiological pH of 7.4; PB: protein binding; Log MW: logarithm of molecular weight; Charge: molecular charge at physiological pH; Oxy surface: oxygenator surface; N passage: the number of matrix passages in the oxygenator (defined as matrix flow rate divided by volume in the ECMO circuit); N drugs: the number of drugs injected in the same ECMO run; drug loss: percentage of drug loss corrected for controls.
